# Purification and Characterization of *Anabaena flos-aquae *Phenylalanine Ammonia-Lyase as a Novel Approach for Myristicin Biotransformation

**DOI:** 10.4014/jmb.1908.08009

**Published:** 2019-09-30

**Authors:** Asmaa M. Arafa, Afaf E. Abdel-Ghany, Samih I. El-Dahmy, Sahar Abdelaziz, Yassin El-Ayouty, Ashraf S. A. El-Sayed

**Affiliations:** 1 Department of Pharmacognosy, Faculty of Pharmacy, Zagazig University, Zagazig 44519, Egypt; 2 Enzymology and Fungal Biotechnology Lab (EFBL), Botany and Microbiology Department, Faculty of Science, Zagazig University, Zagazig 44519, Egypt

**Keywords:** Phenylalanine ammonia-lyase, purification, properties, myristicin, MMDA

## Abstract

Phenylalanine ammonia-lyase (PAL) catalyzes the reversible deamination of phenylalanine to cinnamic acid and ammonia. Algae have been considered as biofactories for PAL production, however, biochemical characterization of PAL and its potency for myristicin biotransformation into MMDA (3-methoxy-4, 5-methylenedioxyamphetamine) has not been studied yet. Thus, PAL from *Anabaena flos-aquae* and *Spirulina platensis* has been purified, comparatively characterized and its affinity to transform myristicin was assessed. The specific activity of purified PAL from *S. platensis* (73.9 μmol/mg/min) and *A*. *flos-aquae* (30.5 μmol/mg/min) was increased by about 2.9 and 2.4 folds by gel-filtration comparing to their corresponding crude enzymes. Under denaturing-PAGE, a single proteineous band with a molecular mass of 64 kDa appeared for *A. flos-aquae* and *S. platensis* PAL. The biochemical properties of the purified PAL from both algal isolates were determined comparatively. The optimum temperature of *S. platensis* and *A. flos-aquae* PAL for forward or reverse activity was reported at 30°C, while the optimum pH for PAL enzyme isolated from *A. flos-aquae* was 8.9 for forward and reverse activities, and *S. platensis* PAL had maximum activities at pH 8.9 and 8 for forward and reverse reactions, respectively. Luckily, the purified PALs have the affinity to hydroaminate the myristicin to MMDA successfully in one step. Furthermore, a successful method for synthesis of MMDA from myristicin in two steps was also established. Gas chromatography-mass spectrometry (GC-MS) analysis was conducted to track the product formation.

## Introduction

**Fig. 1:**
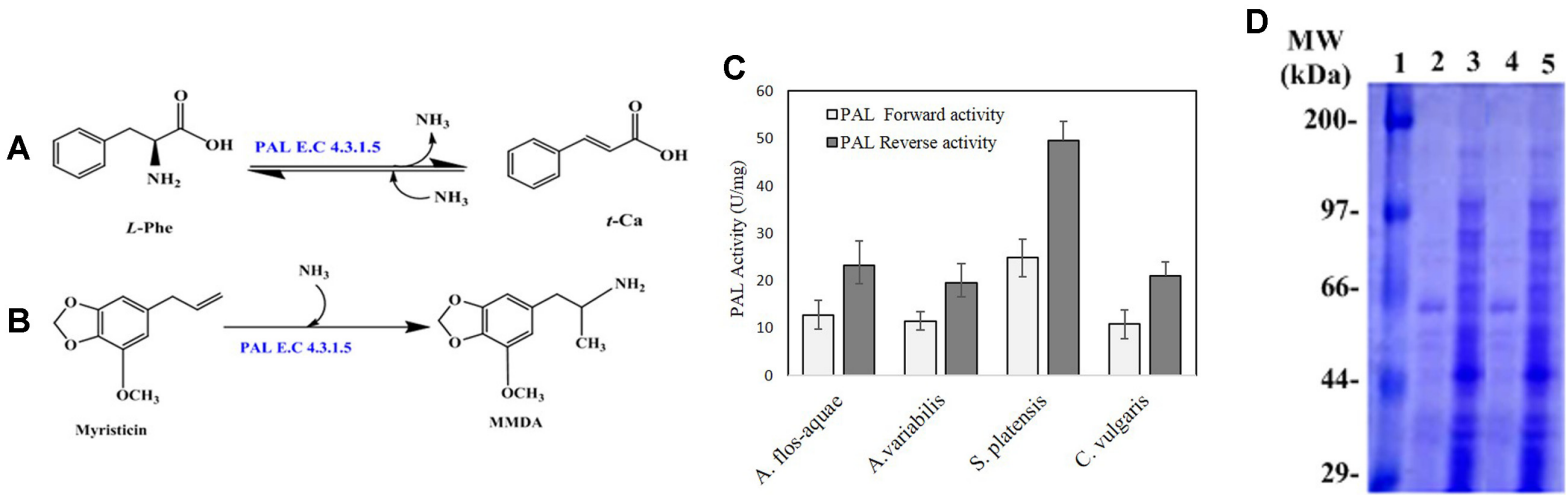
Forward and reverse pathways catalyzed by PAL (A), Proposed biotransformation of myristicin to MMDA by PAL (B), Forward and reverse activities of PAL from different algal isolates (C), SDS-PAGE of the purified PAL from *S. platensis* and *A. flos-aquae* (D). Lane 1, broad range marker (Cat# sc-2361, 6-200 kDa, Santa Cruz Biotechnology, Inc. USA), lane 2 and lane 4 are purified PAL from *S. platensis* and *A. flos-aquae*, respectively, lane 3 and lane 5 are crude extracts of *S. platensis* and *A. flos-aquae*, respectively.

Phenylalanine ammonia-lyase (PAL, E.C. 4.3.1.5) belongs to the ammonia lyases family [1], and catalyzes the deamination (forward) and hydroamination (reverse) reactions of phenylalanine as substrate [2, 3]. The forward reaction involves deamination of *L*-phenylalanine (*L*-Phe) to *trans*-cinnamic acid (*t*-Ca) and ammonia, while, the reversal reaction involves biotransformation of *trans*-cinnamic acid to *L*-phenylalanine [4, 5], as illustrated in Fig. 1A. PAL is widely distributed in plants, acting as a key enzyme in phenylpropanoid pathway [6, 7], controlling the production of secondary metabolites such as flavonoids, coumarins, lignins, stilbenes and phytoalexins [7]. PAL has also been reported found in fungi, yeasts and bacteria [4]. Fungal PAL can utilize *L*-phenylalanine as a carbon and nitrogen source [5, 8]. Bacterial PAL was reported to be implemented in biosynthesis of secondary metabolites as cinnamamide in *Streptomyces verticillatus* [9], 3,5-dihydroxy-4-isopropylstilbene in *Photorhabdus luminescens* and enterocin in *Streptomyces maritimus* [1, 10]. Few studies were documented to the PAL from algae, except *Anabaena variabilis*, *Nostoc punctiforme* [11], *Dunaliella marina* [4] and *Anacystis nidulans* [12]. Animal [4] and human [13, 14] tissues are free of PAL. Recently, there is a growing interest in exploring the biochemical properties of PAL because of its clinical and commercial applications [15]. Chemically modified PAL has been used as a therapy for phenylketonuria (PKU) [9, 16], which is an inborn disorder of phenylalanine metabolism caused by deficiency of phenylalanine hydroxylase (PAH), causing cognitive development loss and mental retardation as a result of hyperphenylalaninemia [17, 18]. Also, PAL has been used for anticancer activity due to its selectivity for phenylalanine by inhibiting the growth of neoplasms in vitro and in lymphoblastic leukemia [4, 15, 19]. Moreover, reverse pathway of PAL meets the great requirements for *L*-phenylalanine in the food and pharmaceutical industries [15]. As an essential amino acid, *L*-phenylalanine [20] serves as a supplement for human nutrition and as a precursor for the synthesis of artificial sweetener aspartame [21-23].

Myristicin (1-allyl-5-methoxy-3,4-methylenedioxybenzene), is a naturally occurring phenylpropanoid derivative [24, 25] found in nutmeg [26], cinnamon, parsley, basil, carrot [27], dill [28] and various plants belonging to the Umbelliferae [29]. Myristicin has various applications in the food and cosmetic industries, in treatment of anxiety, stomach cramps, diarrhea, and cholera in addition to having antibacterial, anti-inflammatory, hepatoprotective, anti-cholinergic [27, 30] and insecticidal [31, 32] activities. Metabolic conversion of myristicin into 3-methoxy-4, 5-methylenedioxy-amphetamine (MMDA) had been studied in rats [33] and humans [34, 35]. MMDA is a potent psychoactive drug [36-38] that has about twice higher activities than those of mescaline [33], with similar activity to 3,4,5-trimethoxyamphetamine (TMA) [39]. MMDA attenuates anxiety and loneliness feelings, suppresses appetite [40] and elevates the mood [35, 41]. MMDA is used for treatment of autism, major depressive disorder, anxiety [42, 43], alcohol dependence, Alzheimer’s, attention-deficit hyperactivity disorder (ADHD), and parkinsonism-dystonia infantile and schizophrenia [43-45]. MMDA has been used as a psychotherapy adjunct [46], as a useful drug for neurosis treatment [35]. Moreover, another study reported at the American Psychological Association’s annual meeting in 2018 that MMDA is used for treatment of social anxiety, depression, and post-traumatic stress disorder [47]. However, production of MMDA is the main technical challenge for its various pharmaceutical applications. MMDA has been synthesized commercially from myristicin by Shulgin [48] and Clark *et al*., [49], however, the price of MMDA is about 17.8 folds higher than myristicin [50]. Thus, searching for alternative novel methods for production of MMDA with higher yield and low price is an objective for many researchers. Production of MMDA via biotransformation of myristicin with PAL seems to be a novel hypothetical approach for higher yield of MMDA, as proposed in Fig. 1B. From literature, PAL of algal sources was reported to have a higher turnover number, catalytic activity and broad substrate specificity, than plant and fungal and bacterial sources of PAL (https://www.brenda-enzymes.org/enzyme.php?ecno=4.3.1.5). Thus, the objective of this work was to purify PAL from different algal sources, in addition to exploring the potentiality of purified PAL to transform myristicin as substrate into MMDA, using one-step enzymatic synthesis. To the best of our knowledge, nothing has been reported on the production of PAL by green algae like *Chlorella vulgaris* and blue-green algae such as *Anabaena flos-aquae* and *Spirulina platensis* except *Anabaena variabilis* var*. kashiensis* (Bharadwaja) [5, 10]. In view of this, the second aim of this research is to discover new microbial sources of PAL production. Thus, our report investigates the extraction, purification, and characterization of PAL from *A. flos-aquae* and *S. platensis*. Moreover, investigation of myristicin biotransformation by purified PAL is the latest aim.

## Materials and Methods

### Algal Strains and Culture Conditions

Fifty microbial isolates (twenty fungal isolates, twenty bacterial isolates, and ten algal isolates) were collected from the microbiology laboratory of the Faculty of Science, Zagazig, Zagazig University, Egypt. The fungal isolates were grown on potato dextrose broth media [51], while the bacterial isolates were grown on nutrient broth media [52]. *Anabaena flos-aquae* and *A. variabilis* were grown on blue-green medium (BG-11) [53], *Spirulina platensis* was grown on Zarrouk medium [54], and *Chlorella vulgaris* was grown on diatom culture medium (DM) [55]. The fungal cultures were incubated for 10 days at 30°C, while bacterial cultures were incubated at 5 days at 37°C. Algal cultures were maintained at 27°C ± 1°C, except *S. platensis*, which was incubated at 31 ± 0.5°C under white fluorescent illumination of 30 to 40 μEm-2s-1 provided by fluorescent tubes (Philips Trulite, Col 82). The cultures were exposed to light: dark photoperiod (12:12) and aerated with air current through an electric pump (20 l/h). The pH of media was adjusted to 7.1 (BG-11), 9.5 (Zarrouk medium) and 6.9 (DM) by 0.1N HCl and /or NaOH using a Jenway 3510 pH meter.

### Algal Harvesting and Extraction of Phenylalanine Ammonia Lyase (PAL)

The algal cells were harvested by centrifugation at 10,000 ×*g* (4°C) for 15 min at mid-logarithmic phase, washed three times with sterile distilled water and the algal masses were stored at -20°C. Twenty grams of frozen algal biomass of each strain were separately pulverized in liquid nitrogen and suspended into 50 ml of 100 mM cold Tris-HCl buffer (pH 8.9), with 0.2% Na_2_-EDTA and 125 μl β-mercaptoethanol. The extracts were centrifuged at 10,000 ×*g* for 10 min at 4°C and the supernatants were used as crude extracts for enzyme assay, protein estimation and purification.

### Phenylalanine Ammonia Lyase (PAL) Assay

The deaminating and hydroaminating activities of the crude enzyme were determined [1, 56, 57] with slight modifications. Briefly, for deaminating activity, assay was performed using 50 μM *L*-phenylalanine, 250 μl of enzyme extract, in 2 ml of 100 mM Tris-HCl buffer (pH 8.9), and incubated at 37°C for 30 min. The reaction was stopped by addition of 500 μl of 1N HCl and the absorbance of cinnamic acid was measured at 270 nm. The concentration of cinnamic acid was calculated with regard to standard curve. For hydroamination assay, the reaction (2 ml) contained 100 mM Tris-HCl buffer (pH 8.9), 200 μl of 1 mM *trans*-cinnamic acid in 5 M NH_4_OH and 200 μl of enzyme preparation and was incubated at 37°C for 30 min. Formation of *L*-phenyl-alanine as a byproduct was quantitatively estimated at 257 nm (Rigol ultra-3660 UV-VIS spectrophotometer). One unit (U) of PAL was expressed by the amount of enzyme that catalyzes the formation of 1 μM of *trans*-cinnamic acid/*L*-phenylalanine per min under optimal assay conditions. The specific activity (U/mg) was expressed by the enzyme activity in unit per milligram of protein.

The concentration of proteins was estimated by Folin’s reagent according to Lowry *et al*. [58], using bovine serum albumin as standard.

### Purification of PAL by Gel-Filtration and Ion-Exchange Chromatography

The crude enzyme from the selected algal isolates was purified using gel-filtration and ion-exchange chromatographic approaches [59-64]. The crude PAL was precipitated with two-fold chilled acetone, and incubated at -20°C for 30 min. The mixture was centrifuged at 6,000 ×*g* for 15 min at 4°C, and the supernatant was decanted. The protein pellets were dissolved in 5 ml of 100 mM cold Tris-HCl buffer (pH 8.9) as described above, followed by centrifugation at 10,000 ×*g* for 5 min at 4°C. Crude extract (3.5 and 8 mg protein/ml) of PAL from *S. platensis* and *A. flos-aquae*, respectively, was applied to a previously equilibrated Sephadex G-200 column (30 cm × 2 cm), with 100 mM cold Tris-HCl buffer (pH 8.9). The column was equilibrated with 100 mM Tris-HCl (pH 8.9) at flow rate 0.5 ml/min, and the fractions (1 ml) were eluted. The activity of PAL and protein content were measured for each fraction. The molecular homogeneity of the most active fractions of PAL were checked by SDS-PAGE, the most active and molecularly homogenous fractions were pooled, collected and concentrated by dialysis (Dialysis Membrane, Size 20, Cat# 546-00051, Wako Chemicals, USA) with 100 mM Tris-HCl buffer (pH 8.9) at 4°C, till reduction of the total volume to 2 ml. The pooled fractions of PAL were further purified by ion-exchange chromatography with DEAE-cellulose. The partially purified PAL was loaded to the top of DEAE-cellulose column previously equilibrated with 100 mM Tris-HCl buffer. The enzyme was eluted on the same buffer with gradient concentrations of NaCl (100-500 mM). The activity of PAL and protein concentration for each fraction were analyzed as described above. The molecular homogeneity of the active fractions was checked by SDS-PAGE analysis, and the molecularly active homogenous fractions were pooled, gathered and concentrated by dialysis, prior to further biochemical analyses.

### Subunit Structure and Molecular Mass of Purified PAL

The subunit structure and molecular mass of the purified PAL from the algal species were determined by the SDS-PAGE and native-PAGE [65, 66], according to Laemmli [66] with slight modifications [51, 64]. The protein samples (50 μl) were boiled in dissociation loading buffer for 5 min, and then loaded into the wells of stacking gel. The gel running was conducted at 100 mA for 40 min (Bio-Rad, Model 2000/200). After running, the gel was immersed in Coomassie brilliant blue stain with gentle shaking at 50 rpm, then, the gel was washed by de-staining. The molecular weight of the appeared protein bands was calculated from the inference of protein ladder (Cat# sc-2361, 6-200 kDa, Santa Cruz Biotechnology Inc., USA).

### Biochemical Properties of Purified PAL

The biochemical properties of the purified PAL such as reaction temperature, thermal stability, reaction pH, pH stability, substrate specificity and effect of various compounds and metals were studied as described previously [51, 62-64]. Michalis-Menten constant (K_m_), maximum velocity (Vmax), turnover number (*k*_cat_) and catalytic efficiency (*k*_cat_/K_m_) are the common kinetic parameters that were determined towards the substrates [60].

### Enzymatic Synthesis of MMDA with PAL Using Myristicin as Substrate

Myristicin was isolated and purified from wild *Daucus pumilus* (Gouan) and its chemical structure was validated from the spectrometric analyses [67, 68]. The biotransformation of myristicin into 3-Methoxy-4, 5-methylenedioxyamphetamine (MMDA) was illustrated in Fig. 1B. The proposed mechanism of the hydroamination reactions of myristicin to MMDA using PAL has been postulated by Lovelock [1]. The enzymatic reaction contained 30 mM myristicin (dissolved in acetone) in Tris-HCl buffer (pH 8.0), and 500 μl of PAL preparation in 5 ml total volume, and the reaction was incubated at 37°C for 1 h with shaking at 120 rpm. Blanks of enzyme free substrate and substrate free enzyme were used as baseline. The reaction pH was adjusted to pH 12 with the addition of 10N NaOH and the product was extracted with diethyl ether three times (20 ml each). Upper phase was combined and concentrated. The concentrated residue was dissolved in 500 μl of methanol and analyzed using gas chromatography-mass spectrometry (GC-MS).

### Chemical Synthesis of MMDA

Chemical synthesis of MMDA was performed by hydrohalogenation followed by amination reactions according to Clark, *et al*., [49] with slight modification. Briefly, hydrohalogenation of myristicin was carried out by addition of 48% of hydrogen bromide (HBr) with stirring at room temperature, then the reaction was terminated by cooling in ice, and the hydrohalogenation product was extracted with diethyl ether. The ether layers were collected, washed with distilled water and concentrated by rotary evaporator at 50°C and 100 rpm. The resultant oil was dissolved in methanol, stirred with 35% NH_4_OH at room temperature to catalyze the amination reaction. After evaporation of the mixture, the resulting oil was dissolved in 10% HC1 and washed with ether. The pH was adjusted to 12 with the addition of NaOH pellets. The aqueous basic solution was extracted with ether, and the pooled ether extracts were evaporated till dryness under reduced pressure. The hydrohalogenation and/or amination reactions were monitored by precoated thin-layer chromatography (TLC) plates (silica gel 60, GF_254_ (60-250 mesh), Merck, Germany) using a solvent system (hexane: methylene chloride (1:1)) and *p*-anisaldehyde sulfuric acid spray according to Gerlacha *et al*. [69], as visualizing agent. The TLC plates were heated for about 5 min at 100°C, and resultant oil from hydrohalogenation and/or amination reactions was analyzed directly by GC-MS.

**GC-MS analysis of synthesized MMDA.** The chemical and enzymatic synthesized MMDA were analyzed by Agilent 6890 gas chromatograph equipped with an Agilent mass spectrometric detector, with a direct capillary interface fused with silica capillary column PAS-5 ms (30 mm × 0.32 mm × 0.25 μm film thickness). A 1 μl sample in methanol was injected to the GC-MS [49], with helium as carrier gas at a flow rate of 1 ml/min. The solvent delay was 3 min and the mass spectrometric detector was operated in electron impact ionization mode with an ionizing energy of 70 e.v. scanning from m/z 50 to 500. The ion source temperature was 230°C and the electron multiplier voltage (EM voltage) was maintained at 1,250 v. The GC was manually tuned using perfluorotributyl amine, the temperature program was started at 70°C then elevated to 150°C at a rate of 15°/min and from 150°C to 250°C at a rate of 25°/min with 6 min hold time. The detector and injector temperature were set at 280°C and 250°C, respectively. The putative names of the target compounds from the spectroscopic data were identified from Wiley and NIST spectral libraries.

## Results and Discussion

### Screening for Phenylalanine Ammonia-Lyase from Different Microbial Sources

Among 50 microbial isolates (20 fungal isolates, 20 bacterial isolates and 10 algal isolates) (Supplementary Data), four algal isolates, namely; *A. flos-aquae*, *A. variabilis, S. platensis* and *C. vulgaris* were selected for their promising yield of PAL. The crude PAL from the four algal isolates displayed visual forward and reverse PAL catalytic reactions on phenylalanine and cinnamic acid as substrates, respectively. The forward activities of PAL from *A. flos-aquae*, *A. variabilis, S. platensis* and *C. vulgaris* were 12.8, 11.5, 24.8, and 10.8 μmol/mg/min, while the reverse activities were 23.3, 19.6, 49.6, and 21.0 μmol/mg/min, respectively (Fig. 1C). These results revealed that *S. platensis* and *A. flos-aquae* displayed the highest PAL activities, thus, the enzyme from both sources has been further purified and characterized comparatively.

### Purification, Molecular Subunit Structure of PAL from Selected Algal Isolates

The PAL was purified from the cultures of *S. platensis* and *A. flos-aquae* by gel-filtration and ion-exchange chromatographic approaches. The purification profile of PAL from *S. platensis* and *A. flos-aquae* was summarized in Table 1. The specific activities of PAL from *S. platensis* and *A. flos-aquae* were increased by about 1.7 and 1.3 folds than their corresponding crude enzymes with an overall yield of 73.3 and 87.1 %, respectively, upon acetone precipitation. By Sephadex G_200_ column, the specific activities of PAL from *S. platensis* and *A. flos-aquae* were increased by 2.9 and 2.4 folds with overall yield of 13.2 and 15.9%, respectively. With the ion-exchange chromatography, the specific activities of PAL from *S. platensis* and *A. flos-aquae* were increased by 4.7 and 3.5 folds comparing to their crude enzymes, respectively. Consequently, the activity of PAL from *S. platensis* was higher than *A. flos-aquae* by 1.3 folds with the last purification step. The active fractions from gel-filtration and ion-exchange chromatography were assessed based on their colorimetric activity and molecular homogeneity by denaturing PAGE. Prior to biochemical characterization, the most active and molecularly homogenous fractions were gathered and concentrated by dialysis with polyethylene glycol.

The subunit structure of PAL from *S. platensis* and *A. flos-aquae* was assessed by gel electrophoresis. SDS-PAGE analysis (Fig. 1D) showed a single protein band of molecular weight 64 kDa, revealing the homogeneity of the purified *S. platensis* and *A. flos-aquae* PAL. The molecular mass of recovered PAL is in coincidence with those reported for cyanobacterial PAL from *A. variabilis* and *Nostoc punctiforme* [10]. However, PAL from *Trichosporon cutaneum* [70] and *Rhodotorula glutinis* [11] showed 79 and 75 kDa, respectively.

### Biochemical Characterization of PAL from *S. platensis* and *A. flos-aquae*

**Fig. 2:**
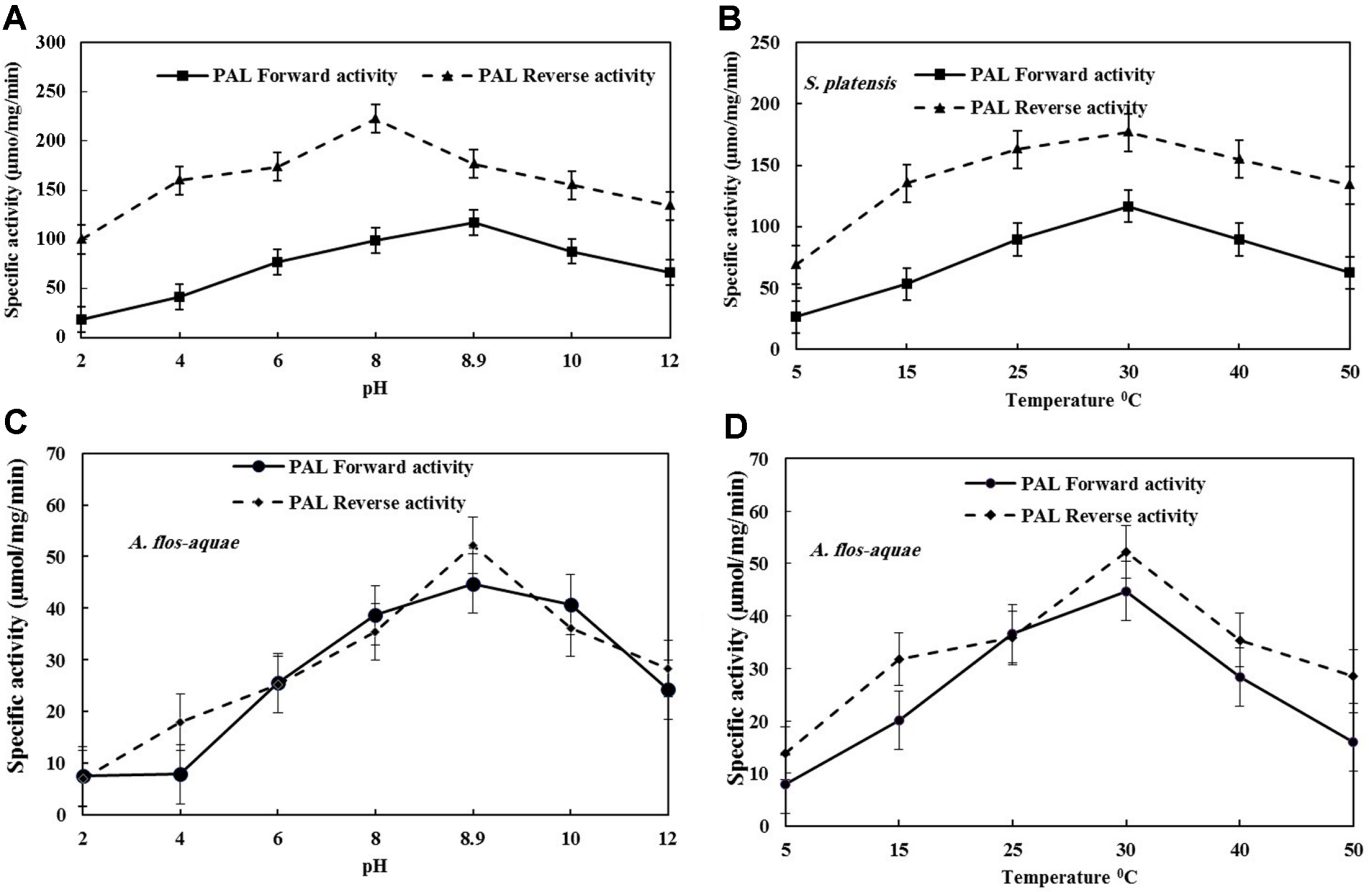
Effect of different pH and temperature on specific activity of purified PAL from *S. platensis* (A and B) and *A. flos-aquae* (C and D), respectively. Each data point shows the average of at least three replicates. The standard errors are represented by vertical bars.

**Optimum reaction temperature and pH.** The effect of reaction temperature and pH on the activity of PAL from both algal isolates was assessed. From the results (Fig. 2), the purified PAL from *S. platensis* had a maximum forward (116.7 μmol/mg/min) and reverse activity (176.7 μmol/mg/min) at 30°C incubation temperature. Also, at 30°C, the maximum activity of *A. flos-aquae* PAL was 44.7 and 52.2 μmol/mg/min for forward and reverse reactions. Being partially consistent, Moffitt *et al*., [10] reported that the optimum temperature of *A. variabilis* PAL was recorded at 40°C, while the optimal activity of PAL of tobacco and sunflower was reported at 35 and 55°C, respectively, and the optimum temperature of PAL of *Rhizoctonia* ranged from 44-46°C [4].

The highest activity of PAL of *S. platensis* was recorded at pH of 8.9 and 8 of forward and reverse reactions, respectively. Moreover, PAL derived from *A. flos-aquae* exhibited maximum activity of forward and reverse reactions at pH of 8.9. The optimal activity of PAL at this pH from both algal isolates were consistent with those reported for PAL production by *A. variabilis* [10] and within the optimum pH range for PAL [4, 8] from various microbial sources. Furthermore, PAL from the yeasts *Trichosporon cutaneum* [70] and *Rhodotorula glutinis* [11] had optimum pH at 8-9, respectively.

**Fig. 3:**
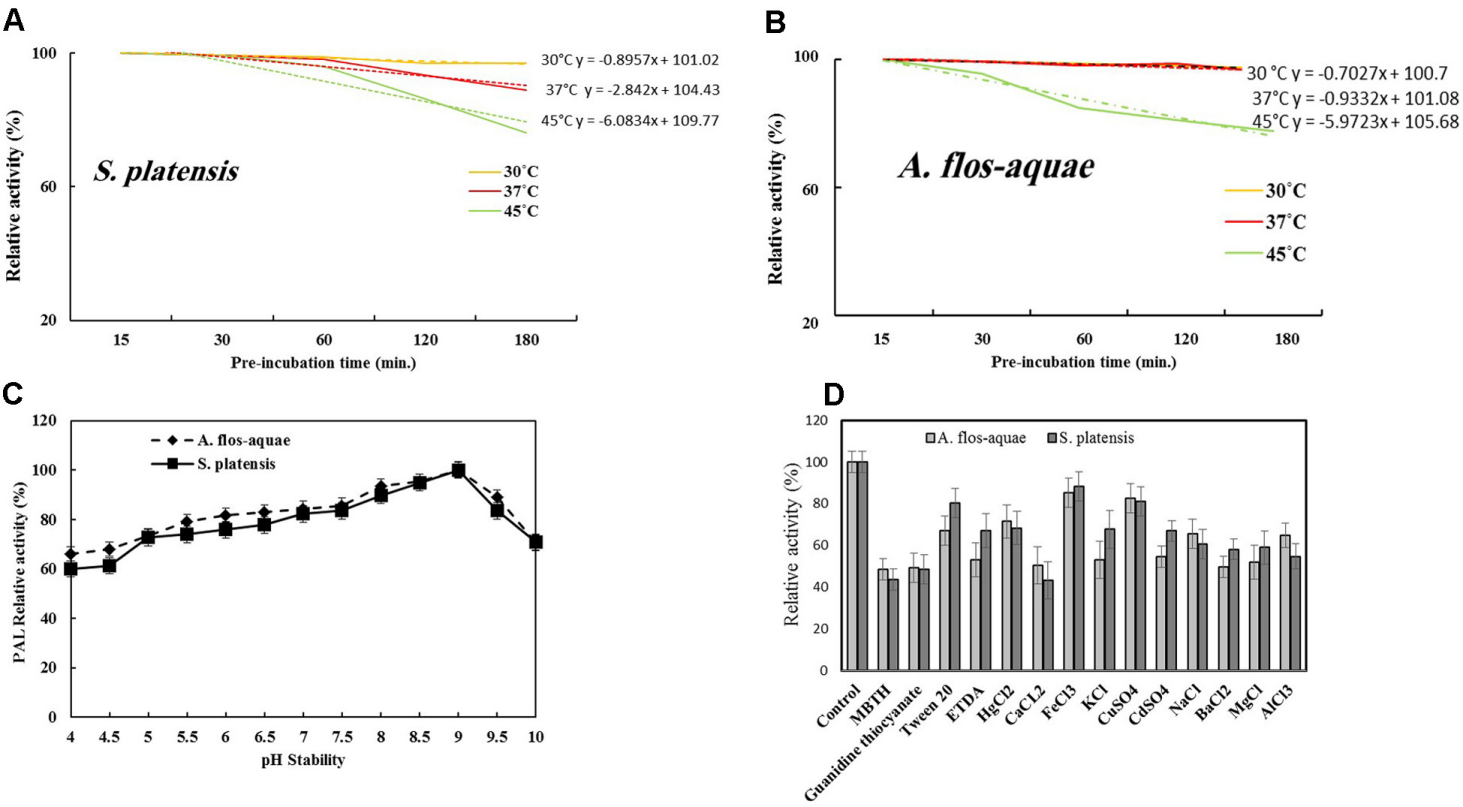
Thermal stability (A&B), pH stability (C), compounds and metals (D) of PAL from *S. platensis* and *A. flos-aquae*, respectively.

**Thermal stability, pH stability, inhibitors and activators of PAL.** The thermal stability of PAL from both algal isolates was determined by pre-incubation of enzyme without substrate at various temperatures (30, 37, and 45ºC). The residual enzyme activity was measured by the forward assay after 15, 30, 60, 120, and 180 min for each temperature degree. The profile of thermal stability of PALs was shown in Fig. 3. The half-life times of PAL from *S. platensis* at 30, 37, and 45°C were 9.5, 3.2, and 1.6 h, respectively. Meanwhile, PAL from *A. flos-aquae* had half-life times of 12, 9.0, and 1.5 h at 30, 37, and 45°C, respectively. Therefore, PAL from *A. flos-aquae* has a relatively higher stability than from *S. platensis*.

The pH stability of PAL was assessed by pre-incubation of enzyme without substrate at different pH ranges (pH range 4-10) using potassium phosphate buffer at 4°C for 2 h, then the residual activity was determined as described above. PALs from both algal isolates displayed a higher stability at pH range 8-9, with dramatic reduction to its activity at acidic pH and higher alkaline pH (Fig. 3C). Zhu *et al*. stated that activity of *Rhodotorula glutinis* PAL was stable at pH range of 6-10 [11].

The effect of various compounds and metals on activity of PAL was estimated by incubating the enzyme with each compound at 1mM final concentration for 2 h at 4°C, then measuring the residual activity by the standard assay. Tested compounds such as 3-Methyl-2-benzo-thiazolinone hydrazone (MBTH), guanidine thiocyanate, Tween 20 and ethylenediaminetetraacetic acid (EDTA) and different metals such as Na^+^, K^+^, Ca^2+^, Cu^2+^, Cd^2+^, Ba^2+^, Mg^2+^, Hg^2+^, Fe^3+^, and Al^3+^ were used. By addition of compounds, the residual activity of PAL from *S. platensis* was maximally reported in presence of FeCl_3_ (88.2%), followed by CuSO_4_ (81.1%) and Tween 20 (80.5%). On the other hand, 85.4% of PAL activity from *A. flos-aquae* remained after incubation with FeCl_3_, followed by 82.7% with CuSO_4_ and HgCl_2_ (71.4%) (Fig. 3D). It was reported that PAL activity from *Cistanche deserticola* was inhibited by Hg^2+^, Zn^2+^, and pb^2+^, while Co^2+^, Fe^3+^, and Cu^2+^ had no significant inhibitory effect [6]. Moreover, Mg^2+^ and Ba^2+^ were reported to be a slight activator for PAL activity [4].

**Substrate specificity and kinetics of the purified PAL.** The specificity of purified PALs from the two algal isolates towards different amino acids had been evaluated based on the standard forward assay. PALs from *S. platensis* and *A. flos-aquae* have affinity only toward tyrosine by 18.32% and 51.15%, respectively, comparing to phenylalanine (Table 2). PALs from the two algal isolates have no activity on other amino acids such as methionine, glycine, asparagines, ornithine, lysine, arginine, alanine, cysteine and valine. These results are also consistent with those reported by MacDonald and D’Cunha [8].

The catalytic and kinetic parameters of the enzyme towards phenylalanine and cinnamic acid were summarized in Table 3. Different concentrations of *L*-phenylalanine and *trans*-cinnamic acid were tested and the activity of enzymes was measured by forward and reverse assay, respectively. PAL from both algal isolates displayed a higher affinity and velocity towards *trans*-cinnamic acid than *L*-phenylalanine. *S. platensis* PAL has a higher turnover number (*k*_cat_) and catalytic efficiency (*k*_cat_/K_m_) (1.25 s^-1^ and 2.03 ms^-1^s^-1^, respectively) than *A. flos-aquae* PAL (32× 10^-2^ s^-1^ and 1.28 ms^-1^s^-1^, respectively) toward *trans*-cinnamic acid as a substrate. Furthermore, turnover number and catalytic efficiency for *S. platensis* PAL (24.4 × 10^-2^ s^-1^ and 14.9 × 10^-2^ ms^-1^s^-1^, respectively) was higher toward *L*-phenylalanine than *A. flos-aquae* PAL (6.6× 10^-2^ s^-1^ and 4.4× 10^-2^ ms^-1^s^-1^, respectively). Whereas, the wild-type cyanobacterial PAL such as *A. variabilis* and *Nostoc punctiforme* had a higher turnover number (*k*_cat_ 4.3 s^-1^ and 1.96 s^-1^, respectively) and catalytic efficiency (*k*_cat_/K_m_ 72.2 ms^-1^s^-1^ and 43.8 ms^-1^s^-1^, respectively) using *L*-phenylalanine as substrate [10] comparing to our algal isolates. Moreover, PAL from *Streptomyces maritimus* had a smaller turnover number (*k*_cat_ 0.0048 s^-1^) and catalytic efficiency (*k*_cat_/K_m_ 2.1 × 10^-3^μM^-1^s^-1^) toward *L*-phenylalanine [71].

### Synthesis of MMDA

**Fig. 4:**
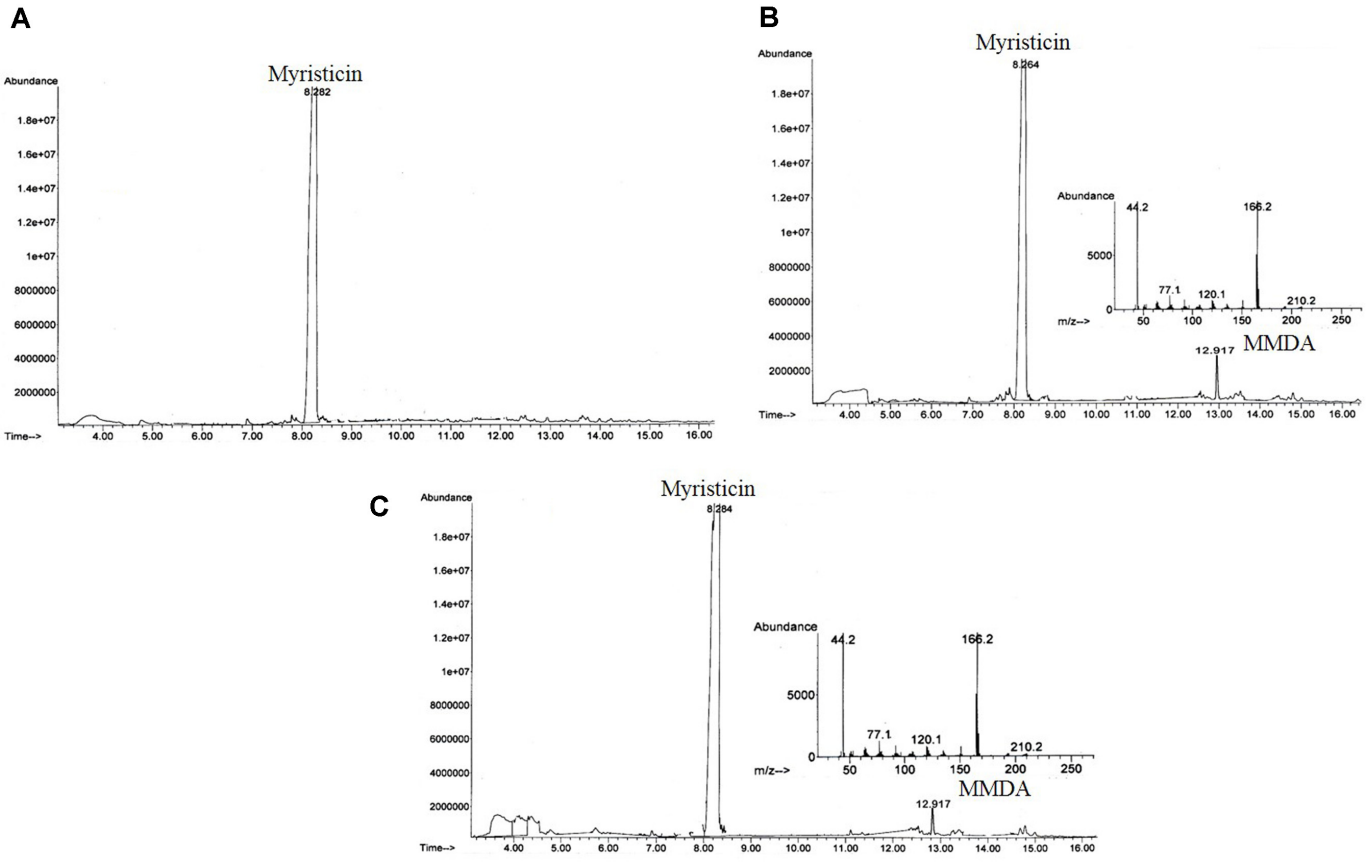
Result of biotransformation of myristicin by PAL. GC chromatogram of blank (A) and reaction of myristicin with PAL from *S. platensis* (B) and *A. flos-aquae* (C) with mass fragmentation pattern of MMDA.

**Enzymatic synthesis of MMDA by PAL from *S. platensis* and *A. flos-aquae*.** The transformation of myristicin with crude PAL of *S. platensis* and *A. flos-aquae* into MMDA was assessed, as described in Materials and Methods. After incubation of the reaction mixture, the enzyme activity was stopped, and the initial concentration of myristicin and developed MMDA was quantified. The enzymatic byproducts were checked by GC-MS analysis using chemically synthesized MMDA as authentic. As shown (Fig. 4), a new peak at 12.917 min for MMDA was observed other than the myristicin peak at 8.26-8.28 min as an enzymatic byproduct of *S. platensis* and *A. flos-aquae* PAL, respectively. The enzymatic yield of MMDA was 15.3 and 10.6% for PAL of *S. platensis* and *A. flos-aquae* using myristicin substrate, respectively. Similarly, PAL from *A. variabilis* [72] and *Rhodotorula glutinis* [20] transformed *trans*-cinnamic acid into *L*-phenylalanine with a yield of about 73% and 70%. In another work, *Rhodotorula glutinis* PAL produced *L*-phenylalanine methyl ester from *trans*-cinnamyl methyl ester in a biphasic system [73]. Furthermore, several reports investigated the hydroamination activity of PAL of microbial [74, 75] or plant origin [76-78].

**Fig. 5:**
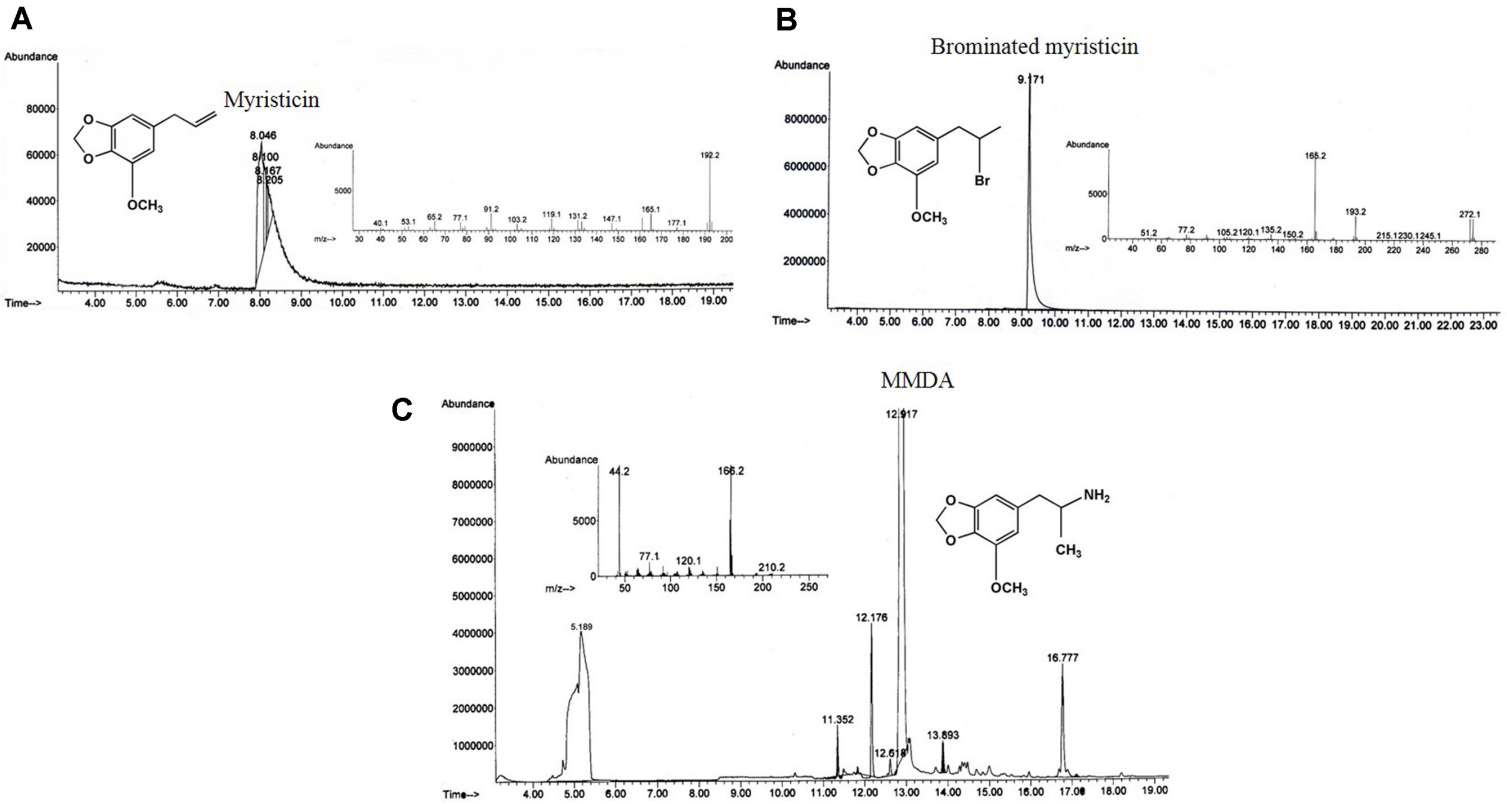
Gas chromatography-mass spectrometry analysis of starting material, intermediate and product during chemical synthesis of MMDA; A, B and C: chromatograms and mass spectrum peaks of myristicin, brominated myristicin and MMDA, respectively.

**Fig. 6:**
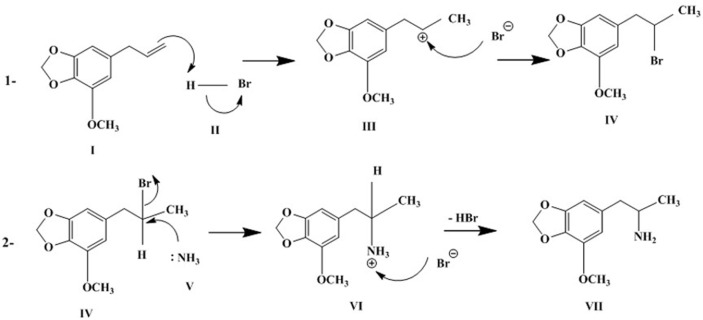
Schematic representation of chemical synthesis of MMDA from myristicin. **1:** hydrohalogenation reaction; **2:** amination reaction I: myristicin; II: hydrogen bromide; III: myristicin carbocation; IV: brominated myristicin; V: ammonia; VI: ammonium cation of MMDA; VII: MMDA

**Chemical synthesis of MMDA.** From the GC-MS analysis (Fig. 5), a successful synthesis of MMDA from myristicin was accomplished. These results were similar to that reported about the synthesis of MMDA derivatives from nutmeg oil [49]. This is the first report dealing with the chemical manufacturing of MMDA from the natural product myristicin via hydrohalogenation and amination reactions. As can be seen from Fig. 6, mechanisms of hydrohalogenation and amination reactions were discussed in detail. Briefly, the electrophilic addition of hydrogen bromide to the side chain, propene group of myristicin gave 2-bromo-1-(3-Methoxy-4,5-methylenedioxyphenyl)-2-propane (brominated myristicin) through the formation of the most stable secondary carbocation. In addition, amination reaction involves bimolecular nucleophilic substitution of ammonia with the intermediate, the brominated myristicin at 9.171 min to produce a desired product, MMDA at 12.917 min. Moreover, the yield of the brominated myristicin and MMDA was 99.22% and 63.63%, respectively.

**Rationality and yield of MMDA from enzymatic and chemical methods.** Biotransformation is the modification of a definite substance to its structurally related product by animal tissues, plants [79] and microorganisms [80, 81], as designated by white biotechnology [82]. Enzymes of microbial origin are preferred over those from animal or plant due to their economic production in a short period of time, high stability under extreme environments and feasibility of their purification [79, 83]. Free enzymes, whole cells and immobilized cells/enzymes are generally used as biological catalysts [80, 82]. High reaction enantio-, stereo- and regioselectivity, minimal byproduct yield, mild reaction conditions and eco-friendliness are the advantages of microbial transformation [82, 84]. Utilization of biological catalysts instead of chemicals in chemical reactions offers great contribution to green chemistry [81, 85, 86] including industrial sectors like pharmaceuticals [80, 81, 87], cosmetics, and food [82]. From the results (Figs. 4 and 5), the actual yield of chemically synthesized MMDA was higher than those of *S. platensis* and *A. flos-aquae* PAL byproducts by about 5.9 and 4.15 folds, using the same concentration of myristicin as substrate. However, the multiple required steps and difficulty of purification of MMDA are the main hurdles that limit the chemical approach. Although enzymatic MMDA has a lower yield, the recovery process makes it more practical and commercially feasible than chemical approaches. Therefore, further optimization of the PAL reaction process for conversion of myristicin into MMDA are ongoing to achieve the maximum yield of MMDA.

In conclusion*, A. flos-aquae*, *A. variabilis, S. platensis* and *C. vulgaris* were used as a new source of PAL. Purification and characterization of PAL from *S. platensis* and *A. flos-aquae* were performed. Furthermore, chemical synthesis of MMDA from myristicin with a yield of 63.63% was carried out. Additionally, MMDA biosynthesis from myristicin by PAL was studied as an eco-friendly route for the first time. Hydroamination of myristicin with the purified PAL from *S. platensis* and *A. flos-aquae* to MMDA was successfully established in a one-step process with a yield of 15.3 and 10.6%, respectively. Consequently, our future work will focus on the over expression and directed mutagenesis of *S. platensis* and *A. flos-aquae* PAL to get deeper insights into this enzyme active site structure and to increase the product yield.

## Supplementary material

Supplementary data for this paper are available on-line only at http://jmb.or.kr.



## Figures and Tables

**Table 1:** Overall purification profile of PAL from *S. platensis* and *A. flos-aquae*

*S. platensis*						*A. flos-aquae*				

Purification step	Total activity (U)	Total protein (mg)	Specific activity (U/mg)	Purification fold	Recovery (%)	Total activity (U)	Total protein (mg)	Specific activity (U/mg)	Purification fold	Recovery (%)
Crude	10222	409.9	24.9	1	100	7598.6	588.9	12.9	1	100
Acetone precipitate	7485	175.6	42.6	1.7	73.3	6619.1	400.1	16.5	1.2	87.1
Gel filtration	1353	18.3	73.9	2.9	13.4	1210.7	39.64	30.5	2.3	15.9
Ion-exchange chromatography	985	9.8	**98.8**	3.9	9.6	850	22.8	**54.7**	4.3	11.2

**Table 2:** Substrate Specificity of purified PAL from *A. flos-aquae* and *S. platensis*

Substrate	*Anabaena flos-aquae*		*Spirulina platensis*	

	Specific activity (U/mg)	Relative activity (%)	Specific activity (U/mg)	Relative activity (%)
Phenylalanine	2.5	100	10.6	100
Methionine	0	0	0	0
glycine	0	0	0	0
asparagine	0	0	0	0
ornithine	0	0	0	0
lysine	0	0	0	0
Tyrosine	1.3	51.2	1.9	18.4
Arginine	0	0	0	0
Alanine	0	0	0	0
Cysteine	0	0	0	0
Valine	0	0	0	0

**Table 3:** Kinetic parameters of PAL from *S. platensis* and *A. flos-aquae* for *L*-phenylalanine (L-PA) and Trans-cinnamic acid (Trans-CA)

Substrate	*S. platensis*				*A. flos-aquae*			
	
	K_m_ (mM)	V_max_ (mM/min)	K_cat_ (s^−1^)	K_cat_/K_m_ (mMs^−1^)	K_m_ (mM)	V_max_ (mM/min)	K_cat_ (s^−1^)	K_cat_/K_m_ (mMs^−1^)
*L*-PA	1.64	0.01	0.24	0.15	1.5	0.01	0.06	0.04
*Trans*-CA	0.61	0.04	1.25	2.1	0.3	0.04	0.32	1.28
