# Supplementary material for: Purification and Characterization of Anabaena flos-aquae Phenylalanine Ammonia-Lyase as a Novel Approach for Myristicin Biotransformation
Source: J Microbiol Biotechnol. 2019 Sep 30;30(4):622–32. doi: 10.4014/jmb.1908.08009 (PMC9728195; doi:10.4014/jmb.1908.08009)
Supplement: Supplementary file 1 [file JMB-30-4-622-supple.pdf]

J. Microbiol. Biotechnol.
https://doi.org/10.4014/jmb.1908.08009

Table S1: Activity of Phenylalanine ammonia lyase from different microorganisms

	Isolate No.	Microbial isolate	PAL forward activity (U/mg)
	1	*Aspergillus terreus*	5.2
Fungi	2	*A. fumigates*	0
	3	*A. flavus*	1.8
	4	*A. parasiticus*	5.7
	5	*A. oryzae*	3.2
	6	*A. niger*	2.0
	7	*A. tamarii*	2.0
	8	*A. carneus*	1.9
	9	*A.sparsus*	0
	10	*A. niveus*	0
	11	*A.ochraceos*	0
	12	*A. flavipes*	0
	13	*A.candidus*	7.5
	14	*Fusarium* sp	6.0
	15	*F. fujikuroi*	4.5
	16	*F. oxyporum*	4.8
	17	*Penicillium crustosum*	3.6
	18	*P. notatum*	3.1
	19	*Humicola* sp	3.1
	20	*Colletotricum* sp	2.6
Bacteria	21	*Bacillus subtilis 1*	1.2
	22	*B. subtilis 2*	2.3
	23	*Bacillus megaterium*	3.5
	24	*Bacillus cereus 1*	5.2
	25	*B. cereus 2*	0
	26	*B. thuringiensis 1*	0
	27	*B. thuringiensis 2*	0
	28	*B. thuringiensis 3*	0
	29	*B. thuringiensis 4*	3.0
	30	*Pseudomonas aeruginosa 1*	3.1
	31	*P. aeruginosa 2*	2.0
	32	*P. aeruginosa 3*	2.8
	33	*P. aeruginosa 4*	1.0
	34	*Pseudomonas sp. 1*	1.8
	35	*Pseudomonas sp. 2*	1.2
	36	*Pseudomonas sp. 3*	1.9
	37	*Pseudomonas sp. 4*	0.9
	38	*Staphylococcus sp. 1*	0.8
	39	*Staphylococcus sp. 2*	0.5
	40	*Staphylococcus aureus*	0.6
Algae	41	*Anabaena flos-aquae*	12.8
	42	*A. variabilis*	11.5
	43	*Spirulina platensis*	24.8
	44	*Chlorella vulgaris*	10.8
	45	*Anabaena* sp. 1	5.2
	46	*Anabaena* sp. 2	5.8
	47	*Spirulina* sp. 1	2.2
	48	*Spirulina* sp. 2	3.8
	49	*Chlorella* sp.1	4.7
	50	*Chlorella* sp.2	5.1
